# Spontaneous Fractures in Long Bones

**Published:** 1910-02-12

**Authors:** 


### Surgery.

### SPONTANEOUS FRACTURES IN LONG BONES.

We are accustomed to regard fractures in long bones as being the result of violence direct or indirect: in the former case the violence is generally severe, but in the latter the degree of severity varies. It is, for instance, a matter of common knowledge that certain forms of fracture can be produced by quite a slight degree of violence, and fractures of the patella and of the neck of the femur afford examples of this. Intracapsular fractures of the neck of the femur are, as is well known, sometimes produced by an injury almost incredibly slight in itself, such as tripping over an inequality in the carpet. But this accident almost always occurs in aged patients, and in them we find a disposing cause to account for the apparent anomaly. In advanced life there is a modification of the arrangement of the lime salts in the body, which we will call, for want of a better term, "a calcareous metastasis." The salts are absorbed from the bones, and particularly from the calcar femorale, which normal acts as a strengthening agent to the neck of the femur, and are laid down in the vescular giving rise to the senile arteritis which is associated with old age. The bone thus becomes brittle, and is unable to withstand even a slight strain.

There are also general morbid conditions in which the long bones fracture at the slightest excuse. This is the case in the disease known as fragilitas ossium. The condition is apparently a congenital one, though little or nothing is known of its causation or pathology. It must not, however, be confounded with mollities ossium, in which the bones become unduly plastic and bend. In fragilitas they become brittle and break, and the probability is that a subject of this disease will have had one or more fractures of each long bone before arriving at adult life. Curiously enough the bones unite readily enough. Here the history of multiple fractures in response to slight violence in the past should make the diagnosis easy.

### Fracture in Malignant Disease.

One condition should always be borne in mind when a fracture is seen in the shaft of a long bone, produced by violence apparently disproportionate to the result, and that is a metastatic deposit of malignant disease in a bone, sometimes quite a considerable distance from the primary. In such cases a thorough routine examination should be made for a primary focus of malignant disease. The following case is interesting in view of these remarks. An old woman, aged 71, was stooping down in her house to lift a moderately heavy object from the floor. She had just succeeded in doing this when she felt something give way in her left leg, and she fell to the ground. She had always enjoyed good health. When she was seen it was quite obvious that she had sustained a fracture at the centre of the shaft of the left femur; the deformity was typical, and crepitus could be obtained. The limb was put up on a Hodgen's splint, but it was found after three weeks of this, when the strapping was about to be re-applied, that there was absolutely no attempt at union. A skiagram was then taken. This had not been done in the first place because it was thought that the condition was so obvious. It was discovered by this means that the ends of the fragments were slightly enlarged, and that the surfaces were irregular. There was no callus at all thrown out. A systematic examination of the patient was accordingly made, and in the left breast a swelling was found which was undoubtedly an atrophic scirrhus. The patient herself was unaware of its presence, as it had never given rise to any symptoms, and she was therefore quite ignorant as to how long it had been growing. It became necessary to amputate the leg, and microscopical examination showed that the bone was invaded by a growth which had all the histological characteristics of a carcinoma mammæ. It seems difficult at first sight to explain a metastatic deposit in the femur from a primary focus in the breast, but the difficulty has been overcome by Mr. Handley's work on the spread of carcinoma along fascial planes.

### Another Illustrative Case.

And yet another case, not quite so dramatic as the first, because the existence of the primary lesion was known when the fracture occurred. The patient was a man, aged 45, who sought advice on account of hæmaturia and pain on micturition. He was examined with the cystoscope, and found to have an inoperable malignant growth of the bladder. He was allowed to get about, and one day, while stooping down, he felt something give way, and he fell to the ground. In this case also it was discovered that he had a fracture of the shaft of the left femur, since it was known that he had a carcinoma of the bladder, metastasis in the femur was suspected, and a skiagram was taken. This displayed a most interesting state of affairs. Not only was there a large growth in the centre of the shaft of the femur at the site of the fracture, but the whole of the ramus of the pubis and ischium had apparently been eaten away by disease, since in the $x$-ray negative this area was simply represented by a light patch. But the curious thing was that although the metastatic changes were so advanced, the patient complained of no symptoms directly referable to them up to the time of the fracture. In view of his general condition, treatment consisted in making the patient as comfortable as his circumstances permitted. He died within two months of the occurrence of the fracture.

These two cases illustrate the statement that it is as important to make a thorough examination of the patient in an apparently simple surgical case as it is in a medical one. The lack of such a precaution in the first case did actually cause the giving of a prognosis which was far from being justified by the subsequent course of the case.

